# The Efficacy of a Personalized mHealth Coaching Program During Pregnancy on Maternal Diet, Supplement Use, and Physical Activity: Protocol for a Parallel-Group Randomized Controlled Trial

**DOI:** 10.2196/31611

**Published:** 2021-11-16

**Authors:** Rozina Nuruddin, Khadija Vadsaria, Nuruddin Mohammed, Saleem Sayani

**Affiliations:** 1 Department of Community Health Sciences Medical College The Aga Khan University Karachi Pakistan; 2 Department of Obstetrics and Gynaecology Medical College The Aga Khan University Karachi Pakistan; 3 Digital Health Resource Center The Aga Khan University Karachi Pakistan

**Keywords:** coaching, compliance, diet, maternal health, mobile health, offspring health, physical activity, pregnancy, supplement use, usability

## Abstract

**Background:**

Adequate intake of macro- and micronutrients and adoption of an active lifestyle during pregnancy are essential for optimum maternal and fetal health and offspring development. Dietary counseling and advice regarding adequate physical activity are integral components of antenatal care. Personalized coaching through the use of mobile health (mHealth) that supports behavior modification is an innovative approach that needs exploration.

**Objective:**

Our primary aim is to assess the efficacy of an mHealth program in improving diet, supplement use, and physical activity during pregnancy. Secondary objectives include evaluation of the program’s effect on maternal and offspring health outcomes and assessment of its compliance and usability.

**Methods:**

A randomized controlled trial was initiated at the Aga Khan University Hospital in Karachi, Pakistan, in January 2020. We aim to recruit 300 pregnant women in their first trimester who have smartphones, do not have comorbidities, and are not taking medications. The intervention group will be trained to use an mHealth app called *PurUmeed Aaghaz*. Through this app, the subjects will report information about their diet, supplement use, and physical activity and will receive personalized advice and three push messages as weekly reminders. The research assistant will obtain similar information from the control group via a paperless questionnaire; this group will receive standard face-to-face counseling regarding diet, supplement use, and physical activity. Data will be collected at enrollment and during four follow-up sessions scheduled 6 weeks apart. Primary study outcomes include improvements in diet (ie, change in mean dietary risk score from baseline to each follow-up), supplement use (ie, changes in mean supplement use score and biochemical levels of folic acid, iron, calcium, and vitamin D on a study subset), and mean duration of reported physical activity (minutes). Secondary study outcomes relate to maternal health (ie, gestational diabetes mellitus, gestational hypertension, pre-eclampsia, and gestational weight gain), newborn health (ie, birth weight and length and gestational age at delivery), and infant health (ie, BMI and blood pressure at 1 year of age). Compliance will be determined by the proportion of participants who complete the 6-month coaching program. Usability will be assessed based on features related to design, interface, content, coaching, perception, and personal benefit.

**Results:**

The study was approved by the Ethics Review Committee of the Aga Khan University in 2017. The recruitment of study participants was completed in September 2021. All follow-ups and outcome assessments are expected to be completed by March 2023 and analysis is expected to be completed by June 2023. We expect the results to be published by the end of 2023.

**Conclusions:**

This study will be an important step toward evaluating the role of mHealth in improving behaviors related to a healthy diet, supplement use, and promotion of physical activity during pregnancy, as well as in influencing maternal and offspring outcomes. If proven effective, mHealth interventions can be scaled up and included in antenatal care packages at tertiary care hospitals of low- and middle-income countries.

**Trial Registration:**

ClinicalTrials.gov NCT04216446; https://clinicaltrials.gov/ct2/show/NCT04216446

**International Registered Report Identifier (IRRID):**

DERR1-10.2196/31611

## Introduction

### Role of Maternal Diet and Micronutrient Supplement Use

Maternal diet serves as a critical prenatal modifiable factor that influences fetal growth and development. Inadequate consumption of macronutrients (ie, proteins, carbohydrates, and fats) and micronutrients, especially folic acid, iron, calcium, and vitamin D, may influence the programming of the offspring’s organs with health consequences during the life course [1]. Evidence suggests strong associations between maternal undernutrition and fetal growth restriction [2]. Consumption of a Mediterranean-type diet rich in fruits and vegetables produces a remarkable decrease in the risk of preterm birth [3,4], hypertension [5], and gestational diabetes mellitus [6]. Adequate consumption of fish and folic acid has beneficial effects on the prevention of pre-eclampsia and gestational hypertension [7, 8].

Poor nutrition during the sensitive phase of the first trimester of pregnancy not only affects growth in the later trimesters and birth weight [9-11] but could also derange epigenetic programming, resulting in long-lasting health consequences [12]. Subtle variations in nutrition during pregnancy and lifestyle factors may influence the risk of noncommunicable diseases without affecting birth weight [13].

The association of micronutrient deficiencies with unfavorable maternal and fetal outcomes is well established. Maternal anemia accounts for low birth weight (12%), preterm births (19%), and perinatal mortality (18%) [14]. Low serum folate levels and absence of folic acid supplementation are associated with preterm births [15]. Similarly, vitamin D and calcium deficiencies are causally linked to pre-eclampsia, gestational diabetes mellitus, preterm delivery, and low birth weight [16].

### Physical Activity During Pregnancy

In addition to diet and micronutrients, physical activity is also known to influence maternal and fetal outcomes. Regular physical activity is recommended during pregnancy for prevention of gestational diabetes and pre-eclampsia [17,18]. The American College of Obstetricians and Gynecologists (ACOG) recommends at least 150 minutes of moderate-intensity aerobic activity every week, or at least 30 minutes on most days of the week, for pregnant women [17,19]. Aerobic activities involve rhythmic movement of large body muscles, such as those in the legs and arms. Moderate intensity refers to moving enough to raise the heart rate and to sweat while being able to talk normally but not being able to sing [20].

### Maternal Health Status in Pakistan

Women of reproductive age in Pakistan are facing a triple burden of malnutrition. A significant proportion of them are underweight (14.4%), overweight (24%), or obese (13.8%) [21]. In addition, there is a high prevalence of micronutrient deficiency, particularly regarding iron (18.2%), calcium (26.5%), and vitamin D (79.7%) [21]. There is also a substantial burden of high-risk pregnancies, causing such complications as gestational hypertension (6.5%) [22], pre-eclampsia (2.4%) [22], and gestational diabetes mellitus (3.3%-17.2%) [23,24]. Pakistan ranks 4th among the top 10 countries with regard to preterm births (16 per 100 live births) [25] and the prevalence of babies with low birth weight (22%) [26].

Of further concern are the suboptimal nutritional indicators for women living in urban areas, where only 42.2% of them have normal BMI compared to 48.5% in rural areas. A significant proportion of these women suffer from undernutrition (12%), overweight (26.5%), and obesity (17.2%) [21]. Also, vitamin D deficiency is more common in urban (83.6%) than in rural (77.1%) settings [21].

Lately, women in Pakistan, in addition to fulfilling their reproductive responsibilities, have started participating in economic activities to support their careers and families. About 17% of women are employed nationwide, and 21% of women who have ever been married are working in the province of Sindh [26]. When they enter pregnancy as nutritionally compromised, these women are found to further neglect their health due to work and other responsibilities [21]. Further, interventions for improving maternal nutrition in Pakistan have been mainly in the form of supplementation of folic acid, iron, multiple micronutrients, calcium, and iodine, as well as balanced protein-energy supplementation [27].

Given the significant magnitude of maternal undernutrition and adverse perinatal outcomes in Pakistan, there is a need to adopt innovative counseling strategies during the critical antenatal period that would regularly remind women to pay attention to their diet and physical activity needs. This would not only reduce the risk of maternal complications, such as pre-eclampsia, pregnancy-induced hypertension, and gestational diabetes, but would also improve the immediate and long-term health of the newborn.

### mHealth Interventions During Pregnancy

The antenatal period represents a major life event when self-motivation to improve diet and lifestyle is often high. This transition to parenthood provides an incredible opportunity, based on the theory of planned behavior [28], because of the willingness to adapt, keeping in mind the expected benefit of having a healthy baby [29]. Hence, communication strategies using mobile health (mHealth) can serve as an awareness tool for desired adjustment in diet and physical activity through positive reinforcement and by offering practical and evidence-based suggestions.

Smarter Pregnancy is one such mHealth intervention launched among the Dutch population in their local language [30]. It was introduced as an online, device-independent, web-based coaching platform for couples during their periconception and pregnancy periods for dietary improvement, folic acid use, and alcohol and smoking cessation [30]. The program consisted of 6 months of interactive coaching through tailored and personalized short text messages and emails; the program showed high compliance (65%) and usability (55%), as well as significant improvement in diet and lifestyle behaviors [30].

### Emergence of mHealth in Pakistan

Being home to a population of over 220 million, Pakistan is experiencing an enormous increase in the digital landscape that parallels the global increase in internet access (45%) and mobile phone subscriptions (96%) [31]. Nationwide, the proportion of internet users has increased by 17% from 2019, reaching 76.38 million in 2020 [32]. According to the recent Digital Pakistan Policy, the Ministry of National Health Services has received support for the promotion of telemedicine and disease prevention information through information and communication tools [33]. With the potential to reach a great proportion of the population at a low cost [31,34], tailored health promotion messages through new delivery modes, such as the internet and mobile phones, have shown promising effects in improving nutrition, lifestyle, and compliance to medication [34-37].

### mHealth-Related Interventions in Pakistan and the Existing Gap

Recently, a few mHealth initiatives have been introduced, such as the Baby+ app for pregnant women in urban areas [38]; the MotherCare app in Swat District [39]; Marham, a private initiative [40]; and Teeko, an Android-based app for improving childhood immunization in rural Sindh [41]. However, there has been no systematic assessment of any locally developed mHealth apps for pregnant women targeted toward improvement in diet, supplement use, and physical activity.

Using an mHealth platform, we intend to empower pregnant women and their health care providers to identify modifiable dietary and lifestyle inadequacies and to receive personalized coaching to address them.

### Study Objectives

#### Primary Objective

Our primary objective is to assess the efficacy of an mHealth coaching intervention compared to standard face-to-face counseling in improving (1) maternal diet by 30%, (2) supplement use (ie, iron, folic acid, calcium, and vitamin D) by 30%, and (3) physical activity by 20%.

#### Secondary Objectives

Our secondary objectives are to conduct the following:

1. Investigate the efficacy of the intervention regarding the following:
  Reducing incidence of gestational diabetes mellitus, gestational hypertension, and pre-eclampsia.
  Improving mean gestational weight gain and mean weight, length, and gestational age at birth.
  Improving mean BMI and blood pressure at 1 year of age.
2. Evaluate compliance (ie, completion of the 6-month program) and usability (ie, design and interface, content and coaching, and perception and personal benefit) of the mHealth app among women in the intervention group.

## Methods

### Target Population

Our target population is pregnant women in their first trimester from the urban area of Karachi, Pakistan.

### Study Goal

Our study goal is to assess the role of the mHealth program in improving the behaviors of pregnant women related to diet, supplement use, and physical activity. In addition, we are interested in assessing whether the maternal, newborn, and infant outcomes differ between those who use the program compared to those who do not.

### Study Hypothesis

We hypothesize that the mHealth coaching program will be effective in improving diet and supplement use among pregnant women by 30% and physical activity by 20% during the study period.

### Study Design

This study is a parallel-group, randomized controlled superiority trial with two groups (ie, intervention and control) having an allocation ratio of 1:1. The trial was registered at ClinicalTrials.gov (NCT04216446) on January 2, 2020. The intervention to be tested is an mHealth program that was developed locally to collect dietary, supplement use, and physical activity information from pregnant women and to provide personalized counseling tailored to the screening information. The control group will receive standard counseling with similar content, but the content will not be personalized and will be delivered through face-to-face sessions.

### Study Setting

The study is taking place at the Aga Khan University Hospital (AKUH) in Karachi, Pakistan. AKUH is a private, not-for-profit, tertiary care hospital, recognized for its trained health care professionals who specialize in providing high-quality compassionate care and for a full range of specialty services [42]. It is also certified by the Joint Commission International Accreditation. The study participants were recruited on a daily basis from the antenatal clinics that are run by trained obstetricians. Most obstetricians, on average, see 25 to 30 pregnant women every day in the clinic.

### Enrollment Criteria

Pregnant women 18 years or older and in their first trimester are eligible to participate if they possess a personal smartphone with internet connection and agree to remain in the study until 1 year following the birth of their baby. Pregnant women on dietary control secondary to the diagnosis of diabetes mellitus; taking medications, such as antiplatelet aggregators, hypoglycemic drugs, or antihypertensive drugs; who have autoimmune, liver, or kidney disease; or who are unable to read and write due to a language barrier will be excluded from the study.

### Sampling Strategy and Randomization

**Figure 1:**
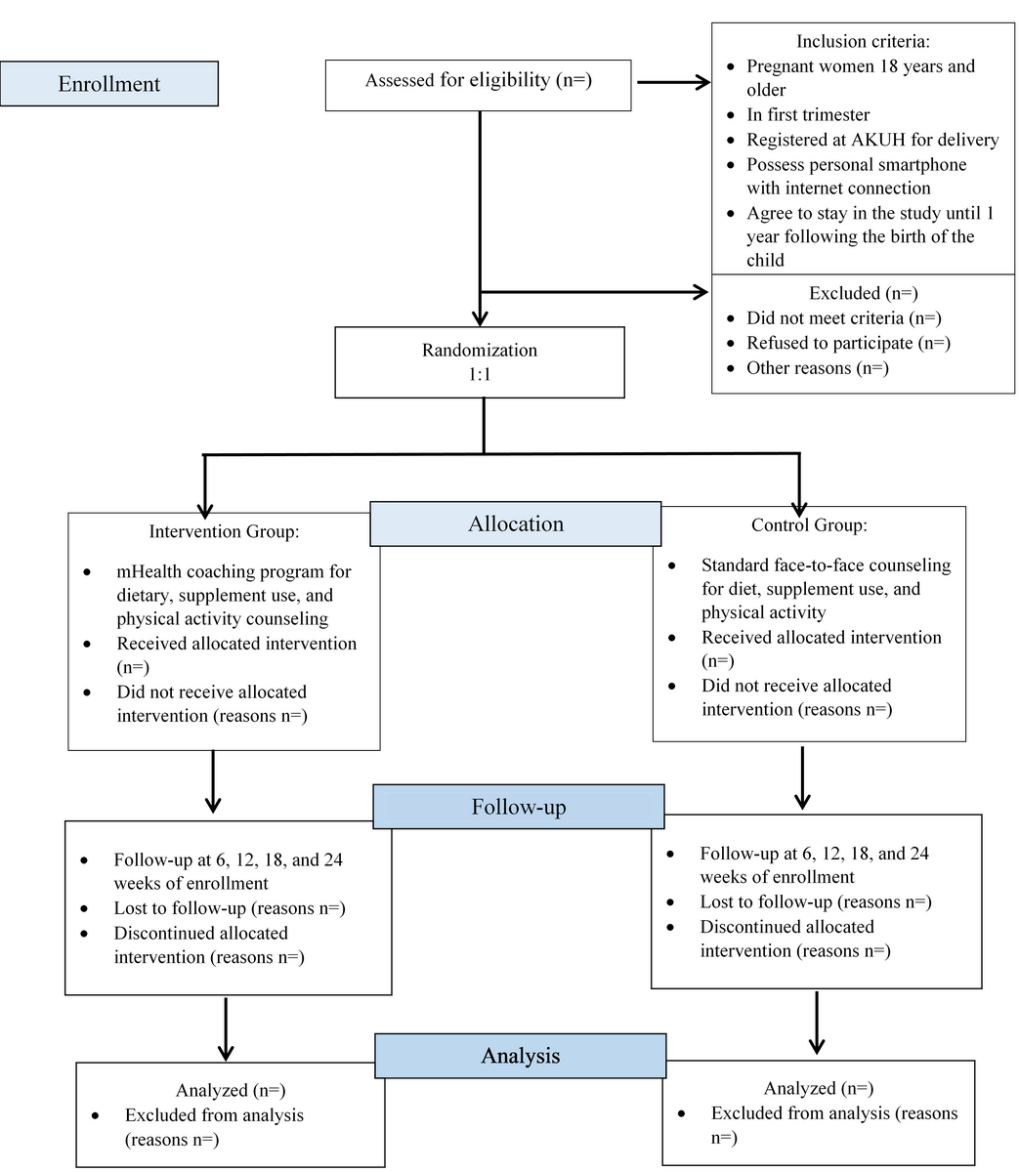
CONSORT diagram of the study. AKUH: Aga Khan University Hospital; mHealth: mobile health.

Participants were identified using a purposive sampling strategy and were randomly assigned to the study groups using simple block randomization (block size of 6). The Clinical Trials Unit at AKUH facilitated the computer-generated randomization and provided opaque, sealed envelopes to the research team to ensure concealment of the assignment (Figure 1).

### Recruitment

At the antenatal clinics, participants were approached to assess their eligibility and to obtain written informed consent. The data collector explained, in detail, the study’s aims and procedures involved in order to facilitate women’s participation and ensure compliance in the study. A signed copy of the consent form was provided to the participants. Enrolled participants were randomized to one of the two groups (Figure 1).

### Web-Based mHealth Program: PurUmeed Aaghaz

**Figure 2:**
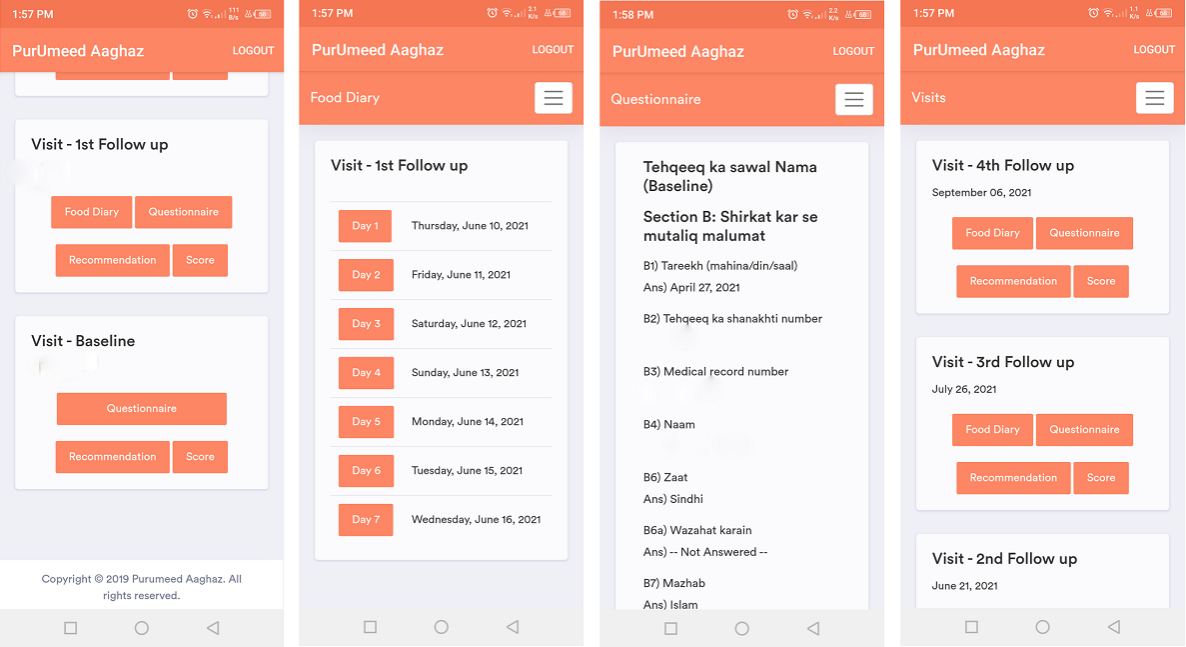
Screenshots of the *PurUmeed Aaghaz* app.

The web-based mHealth program, called *PurUmeed Aaghaz* (a hopeful beginning), was designed and developed locally with the assistance of the Digital Health Resource Center (DHRC) of the Aga Khan Development Network. It is an online, device-dependent app designed to function on Android and iPhone operating systems. It was developed based on the scientific evidence of recommended dietary, supplement use, and physical activity guidelines during pregnancy. Moreover, the coaching platform takes into consideration the theory of planned behavior [43], the theory of self-efficacy [44], the Fogg behavior model [45], and the transtheoretical model for behavior change [46]. Furthermore, the preference, availability, and consumption of locally available items from all the major food groups; supplement use (ie, folic acid, iron, calcium, and vitamin D); and physical activity were taken into account during the development of the app. Based on the participants’ data, the app will generate tailored and personalized counseling messages and recommendations for the pregnant women regarding diet, supplement use, and physical activity. This app will be available to the participants who are randomized to the intervention group as a free subscription, starting from enrollment during their first trimester and for the entire duration of their pregnancy (Figure 2).

### Features of the mHealth Program

The mHealth program has three main features, as discussed in the following three sections.

#### Personalized Recommendations and Dietary Risk Scores

Submission of completed questionnaires by the participants in the first trimester will generate recommendations based on an algorithm. This algorithm will compare participants’ information with the recommended dietary [47], supplement use [48-50], and physical activity guidelines [19] for pregnant women as described in the Counseling for Diet, Supplement Use, and Physical Activity section below. In addition, individualized dietary risk scores for food quantity and diversity in diet will also be generated.

#### Push Messages

Content from the individual coaching sessions will be delivered three times a week in the form of short push messages containing tips and recommendations for diet, supplement use, and physical activity. These messages will be short, simple, and easy to comprehend. Women will be alerted to these messages through notifications on their phones, and these will be available in the app under the “advice” option.

#### Food Record Diary

A built-in food record diary will be available to document information about the food items consumed in a day for breakfast, lunch, dinner, and snacks, along with their portions. Women will be asked to complete the food record diary in their smartphones for a week before their next follow-up, so as to reduce the chance of recall bias. On the follow-up day, the diary will be synced with the data collection tool. Any missing information will be added during the interview.

### Counseling for Diet, Supplement Use, and Physical Activity

#### Diet

Women randomized to the intervention group will receive dietary advice based on the World Health Organization (WHO) guidelines for healthy eating during pregnancy and breastfeeding [47]. We have also consulted guidelines by the WHO for healthy diet [51], the ACOG for nutrition during pregnancy [52], and the Food and Agriculture Organization of the United Nations [53] for the development of content for the push messages. In these messages, consumption of food from six main food groups will be encouraged as follows: bread, cereals, rice, and potato group (6-11 portions per day); vegetable group (at least 3 portions per day); fruit group (at least 2 portions per day); milk and dairy products group (3 portions per day); fish, poultry, meat, and beans group (2 portions per day); and butter, margarine, and oil group (less than 30% of total calories, preferably as unsaturated fats) [51]. In addition, women will be advised to consume three meals and two snacks per day to avoid prolonged periods of fasting and to consume monounsaturated fats, adequate protein, fiber-rich carbohydrates, and at least two servings of omega-3–rich fish per week. Consumption of vitamin A will be encouraged from plant sources as beta carotene. Further, advice will be given to limit intake of carbohydrates with a high glycemic index (eg, fruit juices and sodas).

#### Supplement Use

WHO guidelines for micronutrient supplements during pregnancy will be used to counsel women. These will include daily oral supplements of folic acid (0.4 mg) [48], iron (30-60 mg) [49], calcium (1500-2000 mg) [49], and vitamin D (200 IU) [50].

#### Physical Activity

Based on ACOG guidelines [19], women will be advised to engage in moderate-intensity aerobic physical activity for at least 150 minutes over the week or 30 minutes on most days of the week. Moreover, they will be encouraged to limit prolonged periods of being sedentary [54].

### Control Group

Women randomized to the control group will provide their dietary, supplement use, and physical activity information on a paperless questionnaire administered by the research assistant. Face-to-face counseling will follow, using the bilingual educational brochure from the AKUH on diet during pregnancy and ACOG guidelines for physical activity [19]. A copy of the educational brochure and a paper-based food record diary to be filled in a week prior to their next follow-up will also be provided to women in the comparison group.

### Follow-Up

Follow-ups will be done four times, every 6 weeks: 6, 12, 18, and 24 weeks from the time of enrollment in the first trimester to monitor improvement, if any. For the intervention group, results of each follow-up and their comparison with the earlier one will be displayed on each participant’s personal page of the app. A summary of the individual results will be able to be viewed at any moment by the participant and handed over or shared with their obstetrician by email for further assessment and care. The control group will receive face-to-face counseling at each follow-up.

### Data Collection Tools

#### Screening Questionnaire

A comprehensive questionnaire has been developed to collect data from the participants on sociodemographic characteristics, general food information, anthropometric and blood pressure measurements, biochemical assessments, obstetrics history and supplement use, dietary consumption for the past 7 days recorded as quantity and quality of each food group, and physical activity history for the past 7 days. In addition, intake of savory and fast food, water, tea, coffee, carbonated beverages, and substances such as smoked and smokeless tobacco will be recorded. The questionnaire has been developed after consulting experts and reviewing various dietary assessment tools and programs, such as the 24-hour dietary recall [55], food frequency questionnaires [55], and the Smarter Pregnancy program [56]. The questionnaire has been pretested on 5% of the sample and has been further improved.

#### Food Record Diary

Information about the frequency and portions of food consumed from different food groups over 7 days before the participants’ next follow-up will be collected in the food record diary. The intervention group will report this information in the *PurUmeed Aaghaz* app, while the control group will be required to record this information in a paper-based diary.

#### Biochemical Assessment of Micronutrient Status

In order to validate the information related to dietary intake, every 5th woman enrolled in the intervention and control groups—a subset of 30 women from each group—will undergo a free biochemical assessment of serum iron, ferritin, calcium, and vitamin D at baseline and at the end of the study at the AKUH laboratory. The specimens will be discarded once analyzed by the laboratory.

#### Outcome Assessment Questionnaire

A brief questionnaire has been developed to record information about the study outcomes. The data about maternal and newborn outcomes will be obtained from the medical records, while infant height, weight, and blood pressure will be measured by the trained research assistant.

#### Usability Assessment Questionnaire

**Figure 3:**
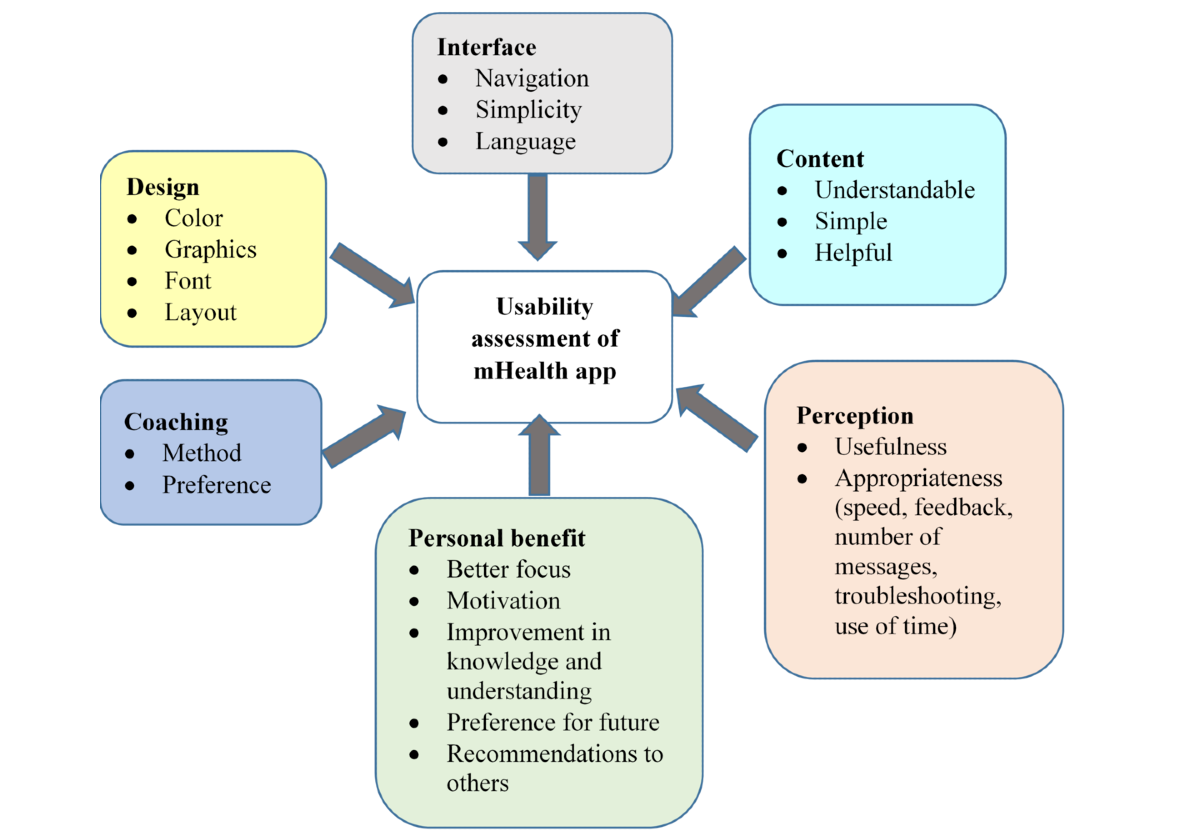
Components of the usability assessment of the mobile health (mHealth) app.

A questionnaire containing 26 questions has been developed to assess the experience of using the mHealth program based on six domains: design, interface, content, coaching, perception, and personal benefit (Figure 3) [36].

### Sample Size Calculation

Sample size has been calculated using OpenEpi (version 3.01). Based on assumptions of an α error of .05, a β error of .2, a 1:1 ratio of exposed to intervention to unexposed to intervention, and improvement in dietary intake and supplement use by 30% in the intervention group from a baseline of 20% [36], a sample size of 45 women in each group will be required. Based on the hypothesized 20% improvement in physical activity from a baseline of 36% [57], our sample size requirement increased to 107 women in each group. To reach 65% compliance [36], approximately 300 women will be needed for this study.

### Expected Primary Outcomes

#### Dietary Intake: Quantity and Quality

Change in dietary intake between baseline and four subsequent follow-up visits 6 weeks apart will be assessed through a questionnaire. Based on consumption recorded in the food diary, data will be collected on the frequency and amount of all kinds of food consumed at each meal, including fruits and vegetables, red meat, white meat, legumes, nuts, eggs, dairy products, added table salt, and drinks (eg, carbonated drinks, fresh juices, and prepared juices). Averages will be calculated to determine the daily intake. Food sizes and amounts will be explained with the help of model utensils (ie, plate, bowl, and glass).

Dietary risk scores, ranging from 0 to 18, will be calculated based on the consumption of food items from six main food groups. Based on portions and diet quality, the score for each food group will be 0, 1.5, or 3 (Table 1) [58]. The total score will be the sum of individual food group scores. The higher the aggregate score, the poorer the dietary quantity and quality, and vice versa. Hence, a score of 18 indicates a highly inadequate dietary intake, a score of 9 indicates a nearly adequate dietary intake, and a score of 0 indicates an adequate diet [58].

**Table 1:** Dietary risk score for six food groups.

Food group and characteristics^a^		Dietary risk score		
		0	1.5	3
**Bread, cereals, rice, and potato**				
	Quantity	6 portions	3 to <6 portions	<3 portions
	Quality	≥50% of whole grains	25% to <50% of whole grains	<25% of whole grains
**Fruits**				
	Quantity	≥2 portions	1 portion	<1 portion
	Quality	Whole fruit consumption	<50% of whole fruits and >50% shakes or fruit juices	Juices and/or shakes only
**Vegetables**				
	Quantity	≥3 portions	1.5 to <3 portions	<1.5 portions
	Quality	>1/3 raw and <2/3 cooked	<1/3 raw and >2/3 cooked	Only in one form
**Fish, poultry, meat, and beans**				
	Quantity	2 portions	1 portion	<1 portion
	Quality	All sources (fish and meat 2 times/week; plant-based protein ≥4 times/week)	Fish >2 or <1 times/week; meat >2 or <1 times/week; plant-based protein 2 to <4 times/week	No fish, no plant-based protein, only meat
**Milk and dairy products**				
	Quantity	3 portions	1.5 to <3 portions	<1.5 portions
	Quality	Milk and dairy products	Milk or dairy products	None
**Oils and fats**				
	Quantity	25% to 30% of total calories	30% to 35% of total calories	>35% of total calories
	Quality	Polyunsaturated	Monounsaturated	Saturated

^a^The quantity and quality refer to the daily consumption.

#### Supplement Use

Supplement use will be assessed by recording the frequency of consumption of folic acid, iron, calcium, and vitamin D in the questionnaire. The frequency will be categorized as daily (7 days per week), often (4-6 days per week), and sometimes (1-3 days per week). Also, a total score ranging from 0 to 12 will be assigned, where use of each supplement will be scored 0 for daily use, 1.5 for less than daily use, and 3 for no consumption (Table 2). The total score will be the sum of each supplement use score and will be monitored at each follow-up.

**Table 2:** Scoring for use of each supplement.

Micronutrient supplement	Frequency of use by score		
	Adequate (0)	In between adequate and inadequate (1.5)	Inadequate (3)
Calcium (1.5-2 g)	Daily	Often or sometimes	Not consumed
Folic acid (0.4 mg)	Daily	Often or sometimes	Not consumed
Iron (30-60 mg)	Daily	Often or sometimes	Not consumed
Vitamin D (200 IU)	Daily	Often or sometimes	Not consumed

#### Physical Activity

Intensity and duration (minutes) of physical activity will be assessed through the questionnaire at baseline and at each follow-up. Walking slowly and household tasks, such as cooking, ironing, light physical work, driving, and washing dishes, will be categorized as mild-intensity activities [59]. Brisk walking; gardening; household chores, such as sweeping, washing, vacuuming, and mopping; actively playing with children; and carrying loads under 20 kg will be classified as moderate-intensity activities [60,61]. On the other hand, vigorous-intensity activities will include running, fast cycling, aerobics, swimming, sports games, or carrying loads over 20 kg [60,61].

### Expected Secondary Outcomes

#### Maternal Health

The following four maternal conditions will be identified through the medical records.

Pre-eclampsia will be defined as new onset of hypertension after 20 weeks of gestation along with proteinuria (ie, a spot urine protein to creatinine ratio of ≥30 mg/mmol) and/or evidence of maternal acute kidney injury, liver dysfunction, neurological features, hemolysis or thrombocytopenia, and/or fetal growth restriction [62].

Gestational hypertension will be defined as new onset of hypertension (ie, blood pressure of ≥140 mm Hg systolic or ≥90 mm Hg diastolic at or after 20 weeks’ gestation) [62].

Gestational diabetes will be defined as diagnosis made by a single-step 75-g oral glucose tolerance test conducted between 24 and 28 weeks of gestation or at any other time, with one or more of the following results: (1) fasting plasma glucose of 5.1 to 6.9 mmol/L (92-125 mg/dL), (2) 1-hour post–75-g oral glucose load of ≥10 mmol/L (180 mg/dL), and (3) 2-hour post–75-g oral glucose load of 8.5 to 11.0 mmol/L (153-199 mg/dL) [63].

Due to the expected low incidence of these conditions and given our small sample size, we shall pool these conditions as adverse maternal outcomes.

Gestational weight gain will be determined from weights recorded during the first, second, and third trimesters. Centiles and *z* scores will be assessed using the international gestational weight gain calculator based on the INTERGROWTH-21st (International Fetal and Newborn Growth Consortium for the 21st Century) Project standards for gestational weight gain [64,65].

#### Newborn Health

At birth, weight, height, and gestational age will be determined from medical records. Birth weight (in grams) will be the first weight of an infant measured soon after birth, ideally within the first hours before significant postnatal weight loss has occurred [66]. Length at birth will be measured from head to toe (in cm). Both weight and length will be adjusted for sex and gestational age at birth and compared with the INTERGROWTH-21st Project reference [67]. Preterm birth will be defined as spontaneous birth before completion of 37 weeks of gestation [68].

#### Infant Health

BMI will be assessed by measuring the weight and length of the infant at their first birthday using standardized equipment (seca 417 length scale and LAICA weight scale, model No. PS3001W1). BMI will be compared with the sex- and age-adjusted INTERGROWTH-21st reference [67]. At the same visit, resting blood pressure will be assessed using the Dinamap VS-900 vital signs monitor (Mindray), with an appropriate infant-sized cuff while the infant is in a state of calm. Two readings will be taken from the left arm, and an average will be calculated [69]. Mean systolic and diastolic blood pressure that is more than the height- and gender-based 90th centile will be considered high risk [70].

#### mHealth Outcomes

Compliance with the intervention will be defined by the percentage of participants who complete the 6-month program [36]. Usability of the mHealth program will be assessed through a usability questionnaire, where items are rated on a 5-point Likert scale with the following responses: 1 (always), 2 (often), 3 (sometimes), 4 (rarely), and 5 (never), or 1 (strongly agree), 2 (agree), 3 (neutral), 4 (disagree), and 5 (strongly disagree).

### Type of Analysis

The data for the study participants will be analyzed based on the group to which they were initially assigned, irrespective of the intervention they received using the intention-to-treat approach [71]. This will preserve the benefits of randomization and will allow us to draw inferences regarding the efficacy of the intervention [71].

### Statistical Analysis

Data will be entered and analyzed using Stata software (version 12; StataCorp LP). Data from the intervention group will be digitalized and uploaded to the app. Categorical variables will be reported as frequencies and percentages. Continuous variables will be reported as means and SDs or medians and IQRs, as appropriate. General characteristics, nutritional change in the form of dietary risk score, supplement use, maternal outcomes, gestational weight gain, birth weight and length, BMI, and blood pressure will be compared using chi-square tests for proportions and *t* tests and Mann-Whitney *U* tests for continuous variables. A mixed-model method will be used to take into account the repeated measurements and correlation while modeling the fraction that scores adequately at each of the follow-up time points. In order to minimize selection bias, we will use multiple imputation models to handle missing data from participants who prematurely leave the study. Possible confounding variables, such as age, BMI, parity, previous history of miscarriage, socioeconomic status, educational status of the women, and occupation, will be assessed and adjusted. Moreover, interactions will be reported if present in the model.

### Data Management, Confidentiality, and Privacy Protection

The intervention group will add data via their smartphones to the mHealth app, which will be protected by log-in ID and password. The data from the program will be uploaded to the web server in real time. The server will be managed by the DHRC team, with access available to the primary person responsible for management of the mHealth program. The data will be handed over to the principal investigator (PI) when requested or upon study completion.

The data from the control group will be gathered on a database built using Microsoft Access. The file will be password protected and access will be limited to the PI and research assistant. The consent forms and the completed food record diaries will be saved in a locker, which will be secured by a lock and key.

### Quality Assurance

To ensure the validity and accuracy of the trial implementation, hands-on training has been given to the research assistant for data collection and for use of the mobile app over the 5 training days using the manual of operations for data collection. Refresher trainings will be conducted every 3 months and as needed. The data collected will be checked every day for possible errors and rectification will be made on the spot. In order to promote participant retention, they will be encouraged to ask any questions. The project PI will closely monitor the study and oversee its smooth implementation. Moreover, regular project meetings will be held for progress monitoring and troubleshooting. Since we do not anticipate changes in the indicators at interim periods of the trial, we intend to conduct intention-to-treat and full analyses at the end of the trial. No adverse events related to the intervention are anticipated.

### Ethical Considerations

Approval for this study has been received from the Clinical Trials Unit and the Ethics Review Committee of the Aga Khan University. Written informed consent will be collected from the study subjects at recruitment. All data collected will be kept strictly confidential and analyzed anonymously. The data shall be used only for research purposes. In case of any changes to the protocol, the Ethics Review Committee will be notified and approval will be sought for amendments.

### Limitations

There are few anticipated limitations of the study. Firstly, the randomization ensures that unknown variables and confounders are randomly distributed among the two groups; however, there could exist residual confounding if not enough data are collected for the confounder in question or if the categories formed are too broad or narrow. Secondly, there could be information bias given that the assessment of behaviors during pregnancy is subjective and, therefore, could lead to differential or nondifferential misclassification. For factors such as substance use and smoking, underreporting can be experienced since these are not socially desirable behaviors. Thirdly, participants may experience recall limitations during the assessment of diet, supplement use, and physical activity. However, in order to minimize the chances of recall bias, a food record diary will be provided to women to document accurate information. Fourthly, the study could experience attrition bias, due to multiple follow-ups and long duration. Fifthly, blinding was not possible owing to the behavioral nature of the intervention. Lastly, as the study will assess the efficacy of the intervention, generalizability will be limited.

### Study Duration and Timeline

We have completed the recruitment and allocation of the participants. Each woman, once enrolled, is expected to remain in the study for a period of 18 months (Table 3).

**Table 3:** Study timeline.

Study steps, assessments, and outcomes			Study period and time point																			
			Enrollment		Allocation		Postallocation															
			Baseline		Baseline		6 weeks		12 weeks			18 weeks			24 weeks			Delivery			1 year of birth	
**Enrollment and allocation**																						
	Eligibility screen	X^a^																				
	Informed consent	X																				
	Randomization			X																		
	Allocation			X																		
**Allocated interventions**																						
	mHealth^b^ coaching^c^			X		X			X				X			X						
	Standard face-to-face counseling			X		X			X				X			X						
**Assessment of independent variables**																						
	Sociodemographics, general food information, and obstetrics history			X																		
	Biochemical assessment information			X		X			X				X			X						
	Diet, supplement use, and physical activity			X		X			X				X			X						
**Measurement of outcomes**																						
	Diet: dietary risk score			X		X			X				X			X						
	Supplement use: biochemical assessment			X												X						
	Physical activity: change in duration			X		X			X				X			X						
	Maternal gestational diabetes					X			X				X			X						
	Maternal gestational hypertension					X			X				X			X						
	Maternal pre-eclampsia					X			X				X			X						
	Maternal weight gain			X					X							X						
	Newborn preterm birth																		X			
	Newborn birth weight and length																		X			
	Infant BMI and blood pressure																					X
	mHealth program compliance and usability															X						

^a^X indicates that the item took place or was measured at this time point.

^b^mHealth: mobile health.

^c^The mHealth coaching intervention occurs continuously over the study period and not just at the specific time points.

## Results

The screening questionnaire was pretested in 2020 on 15 pregnant women (5% of the sample). We completed the study recruitment in September 2021. Of the 300 recruited participants, 22.0% (n=66) were lost to follow-up due to miscarriage, change of hospital, refusal by family to continue, or migration to another part of the country. Of these 66 participants, 64% (n=42) belonged to the intervention group. Of the 234 participants who remained in the study, 72.6% (n=170) have completed their four follow-up sessions. The maternal and newborn outcomes have been assessed for 64.1% (n=150) of the participants, and the infant assessment has been conducted for 2.6% (n=6) of the participants. We plan to complete all follow-ups and outcome assessments by March 2023 and analysis by June 2023. Results are expected to be published by the end of 2023. Trial results will be disseminated through publications and conference proceedings.

## Discussion

Maternal undernutrition and inadequate physical activity in Pakistan pose a serious public health threat. Pregnancy is a critical period when these behaviors directly affect fetal growth and development and influence maternal health in terms of pregnancy-related complications. Considering pregnancy as a window of opportunity, identification and rectification of dietary and lifestyle risk factors could not only provide awareness to women but could also lead to self-actualization leading to behavior change. Addressing dietary insufficiencies is significant not only during pregnancy but also for the health of the offspring throughout the life course. Dietary, supplement use, and physical activity counseling through mHealth has the potential to change behavior by providing tailored and personalized advice. This study will provide the opportunity to test the influence of cost-effective and rapidly evolving mHealth technology on maternal and offspring health in the local context. If proven effective, mHealth will open avenues for improving maternal and child health in Pakistan.

## Appendix

Multimedia Appendix 1 Peer review comments from the funding source.

## Abbreviations

ACOG: American College of Obstetricians and Gynecologists
AKUH: Aga Khan University Hospital
DHRC: Digital Health Resource Center
INTERGROWTH-21st: International Fetal and Newborn Growth Consortium for the 21st Century
mHealth: mobile health
PI: principal investigator
URC: University Research Council
WHO: World Health Organization
