# Supplementary material for: The Efficacy of a Personalized mHealth Coaching Program During Pregnancy on Maternal Diet, Supplement Use, and Physical Activity: Protocol for a Parallel-Group Randomized Controlled Trial
Source: JMIR Res Protoc. 2021 Nov 16;10(11):e31611. doi: 10.2196/31611 (PMC8663618; doi:10.2196/31611)
Supplement: Multimedia Appendix 1 [file resprot_v10i11e31611_app1.pdf]

Aga Khan University
University Research Council Funding
Fourth Cycle 2016

**Reviewers and Members Comments**

| | |
|---|---|
| **URC Project ID: 164003CHS** | **Amount Requested: Rs. 24,527.98/-** |
| **Principal Investigator: Dr. Rozina Nuruddin** | **Department: CHS** |
| **Project: Effectiveness of personalized m-Health coaching program during pregnancy on maternal diet and offspring health: A parallel group randomized controlled design.** | |

**Reviewers' comments:**

- The secondary objectives of the study are too ambitious to be accomplished through the said intervention. The authors are requested to review the secondary objectives carefully and to state the possibility of the change for each secondary outcome.

  The first two secondary objectives have been studied in the Dutch population (see proposal reference30 which is also attached). Based on this experience, as well as our previous nutrition and dietary pattern studies, we are convinced that it is feasible to investigate the compliance and usability of the m-Health intervention amongst 2 x 180 participants. In fact, compared to other nutrition and lifestyle studies in other target groups, this is a very large sample size. We understand and agree that it is not very likely with this sample size to show significant differences in maternal and pregnancy outcomes and child vascular health (our secondary objectives 3-5). However, the aim of studying these secondary objectives is to show a trend and to provide data for a larger trial focusing on these as primary outcomes.

- It seems that the text messages that will be sent would be generic and not customized to individual mothers. If that is the case, then not sure if the title 'personalized m-health coaching program' is justified.

  The extensive and complex algorithm of the m-Health program is built based on decision trees, in which personal conditions as well as personal behaviors are included. In this manner, the program is personalized for the most important determinants: pregnancy state, age and diet. It would generate customized messages based on individual's needs as determined by the assessment scores at baseline and at each follow-up.

- Would be good to include reference of generic m-health counseling based studies. Current references are of personalized messaging base studies.

  Additional references have been added as advised and highlighted in the text on page 3 and in the reference list.

- Prof. Regine Steergers-Theunissen is already conducting research on this topic in the Dutch population.

  Prof. Regine is closely involved in this project because she developed this m-Health program and tested it in a survey and ongoing randomized controlled trials in the Dutch population. Her expertise and close involvement along with that of our local e-health team makes this project highly feasible. This is a major strength of our project.

- Plans on how the study subjects will be tracked are not clear. Plans on how website based follow up will be conducted are not clear.

  The study participants will be identified from amongst the pool of the pregnant women enrolled at the Aga Khan University, Hospital for antenatal care and delivery. They will be

called for their routine antenatal follow-up visits at AKU outpatient clinics and they will be tracked throughout the study period at their follow-up visits.

For the intervention group, the web-based m-health program will automatically deliver dietary counseling content and will ask for filling of follow-up dietary assessments. The non-intervention group will be followed up at the routine antenatal visits for filling of the dietary screening questionnaire on paper rather than through web.

- Using technology for sustainable interventions is the path for future interventions. We need to be clear in our understanding the limits of what it will achieve. The authors are over-ambitious in their secondary objectives.

  The concern regarding the secondary objectives has been answered above.

- Consider having an arm with customized messaging, based on few algorithms.

  This study is based on customized/personalized messaging. We will use total dietary risk scores (DRS) and scores for the individual risk factors as effect measures.

- Have generic text messaging worked for improving maternal nutrition. Give references.

  So far there are no evidence based E-Health programs on maternal nutritional intervention before and during pregnancy. This tool is unique and we can therefore only refer to our own reference 30 which reports that generic text messaging on folic acid use and fruit and vegetable intake shows significant improvement among the intervention group.

- Data collection methodology would be different in intervention vs control arm (computerized vs manual). This is not ideal. How the data for various study outcomes would be obtained can be deferred more thoroughly.

  Due to the nature of intervention, the data for the intervention and control group cannot be collected similarly for dietary status assessment and for compliance and usability of the application. However, information about the maternal and offspring health for the secondary outcomes will be collected in the similar standardized fashion.

- The proposal is based on a good concept but needs review by the authors. This is an effectiveness clinical trial. In the primary outcome authors want to see if M-health intervention will make an improvement in nutrition. However, the authors are not making a baseline assessment of the nutritional intake and frequency. Recommend this should be considered. In secondary outcomes authors are considering an effect on pre eclampsia, gestational hypertension. They should consider adding calcium supplementation. The sample size calculation does not specify the different nutrients being used and the effect of these nutrients on outcomes.

  The reviewer may have overlooked that the Dietary Risk Score (DRS) will be determined at the baseline and monitored for change at the follow-up visits at 6, 12, 18 and 24 weeks after the enrollment. This will be done for both the groups as described in the methodology section. Adding calcium supplementation is a good suggestion and we will program an opportunity for compliance on calcium intake. Furthermore we agree that it will not be feasible to show significant effects on these outcomes but it may be possible to detect some trends. For this reason, we have defined them as secondary outcomes.

- Within nutrition it does not specify the effect of each nutrient on sample size estimation.

  Our primary outcome measures is to assess the change in dietary intake and supplement use (folic acid, iron, calcium and Vitamin D3) between baseline and the follow-up visits for

intervention and control groups through calculation of dietary risk score (DRS). The latter will be based on consumption of food items from six main food groups (bread/wheat, diary product, vegetables, fruits, meat and fish). Hence, sample size calculations are based on assumption of improvement in dietary intake by 30% in the intervention group from a baseline of 20% and are not based on individual nutrient intake.

- The reporting of changes in outcomes will probably be though mean change in score rather than proportion. While the concept of using m-health to improve maternal nutrition is good, It would be suggest that a proof of concept study should be done first to show if text messages influence the practice with regards to maternal diet.

  A large survey of around 1800 users of the m-Health program in the Dutch population has already shown significant improvement (please see reference 30 in the proposal by van Dijk et al 2016). Hence, our study aims to examine the associations in the local context.

- Once this association is established, then a bigger study can be conducted to show its impact on fetal, new born and maternal health.

  We agree that the results of this research project will be important for designing a larger impact study on fetal, child and maternal health outcomes.

**GRC comments:** Revision

Adopt a Dutch program for Pakistani pregnancies and through this smart mobile phone will access theses application so basically it's a mobile base study and get information. The questions are how many researchers the PI wants to run this program

Role of each researcher and collaborator is stated on the GRC form and their individual contribution is indispensible for the project.

and once the program is done than your program should be that much smart so the user should easily use it

This will be judged by investigating the compliance and usability of web-based m-Heath coaching program.

and the other is in which population is your target but vast majority moms don't have literacy to use it and interact with internet based applications.

The eligibility criterion for this study includes pregnant women who have personal smart phones or mobile phones with internet connections. Hence, its results would be generalizable to the population that interacts with internet based applications and not to illiterate population with no access to internet-based application.

Research question should be well addressed for our customized population.

The intervention will be customized to the local needs of the population and in local language.

The m-health checklist is also available online. Sustainability is also a issue. When your population is not literate than how you can convince them. The innovation is not there. Innovate to the population please discuss your sustainability, how many operators you, when your application is ready how many players you need. Innovation and sustainability should be included and it has to be useful for local population. Come up with the justification that how its useful for them. The population in which you're taking the data must check first that those have the smart phone or not. Do you have any data on it state that. Checklist is also included with the comments.

The innovation in this research proposal is its customization according to the local needs of the population and in the local language.  If the project shows significant improvement in our outcome measures, it would be implemented on a larger scale in the clinical setting.
